# Is case triaging a useful tool for emergency surgeries? A review of 106 trauma surgery cases at a level 1 trauma center in South Africa

**DOI:** 10.1186/s13017-018-0166-5

**Published:** 2018-01-24

**Authors:** Sharfuddin Chowdhury, Andrew John Nicol, Mahammed Riyaad Moydien, Pradeep Harkison Navsaria, Luis Felipe Montoya-Pelaez

**Affiliations:** 1 Consultant and head of Trauma Surgery, King Saud Medical City, 7790 Al-Imam Abdul Aziz Ibn Muhammad Ibn Saud, Ulaishah, Riyadh, 12746 Kingdom of Saudi Arabia; 2 Head and Director of Trauma Centre, Groote Schuur Hospital and University of Cape Town, Cape Town, South Africa; 3 Groote Schuur Hospital and University of Cape Town, Cape Town, South Africa; 4 Deputy Director of Trauma Centre, Groote Schuur Hospital, and University of Cape Town, Cape Town, South Africa; 5 Department of Anaesthesia, Groote Schuur Hospital and University of Cape Town, Cape Town, South Africa

**Keywords:** Emergency surgery case triage, Trauma surgery, Postoperative complication, Outcome

## Abstract

**Background:**

The optimal timing for emergency surgical interventions and implementation of protocols for trauma surgery is insufficient in the literature. The Groote Schuur emergency surgery triage (GSEST) system, based on Cape Triaging Score (CTS), is followed at Groote Schuur Hospital (GSH) for triaging emergency surgical cases including trauma cases. The study aimed to look at the effect of delay in surgery after scheduling based on the GSEST system has an impact on outcome in terms of postoperative complications and death.

**Methods:**

Prospective audit of patients presenting to GSH trauma center following penetrating or blunt chest, abdominal, neck and peripheral vascular trauma who underwent surgery over a 4-month period was performed. Post-operative complications were graded according to Clavien-Dindo classification of surgical complications.

**Results:**

One-hundred six patients underwent surgery during the study period. One-hundred two (96.2%) cases were related to penetrating trauma. Stab wounds comprised 71 (67%) and gunshot wounds (GSW) 31 (29.2%) cases. Of the 106 cases, 6, 47, 40, and 13 patients were booked as red, orange, yellow, and green, respectively. The median delay for green, yellow, and orange cases was within the expected time. The red patients took unexpectedly longer (median delay 48 min, IQR 35–60 min). Thirty-one (29.3%) patients developed postoperative complications. Among the booked red, orange, yellow, and green cases, postoperative complications developed in 3, 18, 9, and 1 cases, respectively. Only two (1.9%) postoperative deaths were documented during the study period. There was no statistically significant association between operative triage and post-operative complications (*p* = 0.074).

**Conclusion:**

Surgical case categorization has been shown to be useful in prioritizing emergency trauma surgical cases in a resource constraint high-volume trauma center.

## Background

Trauma is an epidemic in South Africa [1]. The significant number of our emergency general surgical cases is injury related. These are potentially life-threatening, and urgent surgical intervention is required to reduce mortality and morbidity.

Emergency surgical cases are admitted to health institutions in an unplanned and unscheduled manner. Patients usually present with acute surgical conditions that require prompt and focused treatment to avoid increased morbidity and mortality. Currently, no specific national or provincial guidelines exist in South Africa for the categorization or triaging of emergency surgical cases. In the current climate of shrinking elective theater time and increasing surgical waiting time, the present focus is to decrease waiting times by addressing issues that have a detrimental effect on overall theater efficiency. However, emergency surgical caseloads form a significant and increasing percentage of all patients utilizing theater facilities. Finding ways to manage better this category of patients should result in more efficient utilization of available theater time without impinging on the throughput of cases on elective slates. Given this, a policy document or guideline was introduced at Groote Schuur Hospital (GSH) for use in the categorization and prioritization of emergency surgical cases before operative surgical management. At GSH, the Groote Schuur emergency surgery triage (GSEST) system is based on similar principles to the Cape Triaging Score (CTS) [2]. The color coding categories are similar, but the definitions and emphasis relating to levels of surgical acuity, and urgency for operative intervention differ. The surgical team admitting a patient after consultation with the anesthetist handles the initial categorization of the case.

The study aimed to look at the effect of delay in surgery after scheduling based on the GSEST system has an impact on outcome in terms of postoperative complications and death.

## Methods

The University of Cape Town (UCT) Human Research Ethics Committee (HREC) approved the study. We conducted a descriptive, non-interventional, observational study based on the prospective analysis of data collected during 1 December 2013 to 31 March 2014. All adult (age > 13 years) acute trauma admissions presenting with penetrating or blunt chest, abdominal, neck, and peripheral vascular injury who underwent surgery were included. Burns, isolated head, orthopedic, hand, and maxillofacial trauma patients were excluded as the respective specialties managed these patients. Collected data included: patient demographic details, mechanism of injury, time of injury, Injury Severity Score (ISS), time of booking the case, triage color code, the delay from scheduling to the start of the case, reasons for the delay, and outcome regarding postoperative complications and death.

Emergency cases were scheduled according to priority as red, orange, yellow, and green for operation (Table 1). Prioritization was done by the attending trauma surgeon based on patient’s hemodynamics after discussion with the anesthetist.

**Table 1:** Groote Schuur emergency surgery triage (GSEST) system

Icon	Case category	Parameters
Red	Immediate	Immediate life-saving operation, resuscitation simultaneous with surgical treatment, e.g., resuscitative laparotomy, ruptured aortic aneurysm threatened airway, cord prolapse, fetal bradycardia
Orange	Expedited	Operation as soon as possible after resuscitation (within 1 to 2 hours), e.g., ruptured ectopic pregnancy, leaking aortic aneurysm, cranial decompression, positive DPL in multiple traumas, threatened limb, emergent fetal concern
Yellow	Urgent	Operation within 6 h of booking, e.g., compound fractures, appendicitis, incarcerated hernia/intestinal obstruction, EUA for non-accidental injuries
Green	Emergent	Operation not immediately life or limb saving but have to be done within 24 h of booking, e.g., ORIF of simple fractures, bleeding hemorrhoids, I&D abscess
Blue	Scheduled	Semi-urgent cases, to be done within 72 h. Operation during in hours on next available slate if possible

The red cases are an immediate priority (resuscitation with immediate operation cases). These cases usually present with hemorrhage or bleeding, requiring instant, life-saving surgical hemostasis. The orange categories are a very urgent priority (potentially life/limb threatening pathology) that should be done within 2 h. The yellow groups are an urgent priority (significant pathology) that should be done in 6 h, and the green cases are a delayed priority (minor injury/illness) that should be done within 24 h. Green cases become yellow after 24 h. Yellow cases become orange after 6 h. Booked cases are assessed on an ongoing basis and re-categorized as required. Post-operative complications were categorized according to the updated Clavien-Dindo classification of surgical complications [3, 4].

Categorical variables were evaluated using frequency tables. Associations between categorical variables were determined by contingency tables and chi-square tests of association. Numerical variables were evaluated with summary statistics (median, interquartile range, etc.). Relationships between numerical and categorical variables were determined using non-parametric tests: Mann-Whitney in instances where the categorical variable has two categories and Kruskal-Wallis otherwise. A *p*-value of < 0.05 was considered as statistically significant.

## Results

One-hundred six patients underwent surgery during the study period with a higher male to female ratio of 14:1. The median age was 26 (IQR 22–32) years. One-hundred two (96.2%) cases were related to penetrating trauma. Stab wounds comprised 71 (67%) and gunshot wounds (GSW) 31 (29.2%) cases. On presentation 82 (77.4%), patients were admitted with ISS of greater than 15 (5–48). Trauma surgical procedures included laparotomy (48), vascular (27), subxiphoid pericardial window (13), thoracotomy (4), and sternotomy (1). There were also 13 other relatively non-urgent procedures such as debridement, removal of Foley’s cath used for penetrating neck wound tamponade, skin grafting for fasciotomy wounds, etc. Of 106 cases, 6, 47, 40, and 13 cases were booked as red, orange, yellow, and green, respectively. The median delay for green, yellow and orange cases was within expected time. The red patients took unexpectedly longer (median delay 48 min, IQR 35–60 min) to reach the theater (Table 2).

**Table 2:** Operative triage vs. delay (min) from booking to operation

Color code	Expected time to start surgery	Number of cases	Median delay (min)	IQR
Red	Immediate	6	48	35–60
Orange	< 2 h	47	120	53–185
Yellow	< 6 h	40	213	113–300
Green	< 24 h	13	110	65–265

The main (49.1%) reason for the delay in starting the trauma cases was a priority of another emergency (general surgical, gynecological, etc.) booked in that category. Out of six red cases, three were delayed due to unavailability of theater or unable to open an additional theater immediately at that moment (Table 3). One red patient died who presented with a thoracic GSW was unstable on presentation and needed an emergency room thoracotomy and delay to shift to the theater was 55 min after booking.

**Table 3:** Operative triage vs. Reasons for the delay

Cause	Red (N)	Orange (O)	Yellow (Y)	Green (N)	Total (N) (%)
No theater available	3	26	11	2	42 (39.6%)
Priority of other case	0	16	26	10	52 (49.1%)
Delay in shift to theater	3	5	3	1	12 (11.3%)
Total	6	47	40	13	106 (100%)

*N* Number of patients

Thirty-one (29.3%) patients developed postoperative complications (Table 4). Among the booked red, orange, yellow, and green cases, postoperative complications developed in 3, 18, 9, and 1 cases, respectively (Table 5). There was no statistically significant association between operative triage and post-operative complications (*p* = 0.074).

**Table 4:** Types of complications according to Clavien-Dindo classification

Clavien-Dindo Grading	Postoperative complications (number of patients)
I	Wound sepsis (5), Ileus (1)
II	Pneumonia (1)
IIIa	Loculated haemothorax (1)
IIIb	Empyema of chest (3)
IVa	Acute kidney injury (6), respiratory failure (9)
IVb	Multiorgan dysfunction (3)
V	Death (2)
Total	31

**Table 5:** Operative triage vs. complications (yes/no)

Color code	Complications	No complications	Total
Red	3	3	6
Orange	18	29	47
Yellow	9	31	40
Green	1	12	13
Total	31	75	106

## Discussion

Emergency surgical cases differ from elective cases that are planned. During planned or elective operation the length of surgery, hospital stay, and morbidity and mortality can be predicted or inferred. Conversely, emergency surgical cases present in an unpredicted fashion. Prompt, efficient and appropriate treatment of emergency surgical cases is a cost-effective strategy that has the potential to reduce the length of hospital stay, use of high care and intensive care facilities as well as the use of laboratory and other investigative procedures.

The concept of triage is imperative to manage multiple emergency cases with competing and diverse needs. Deficiency of a triage system leads to longer waiting times, poor clinical risk assessment, and management with subsequently increased morbidity and mortality [2]. An efficient triage system minimizes the risk to the patient and promotes effective use of available resources. The rationale and practice of triaging in mass casualty incidents are accepted worldwide [5]. Different triage systems exist for prehospital assessment and in-hospital casualty triage [6–8], yet no widely accepted methods exist for theater bookings. Triage systems should be implemented for the care of surgical emergencies [5] including trauma surgery. Early management of trauma cases is pertinent, as a delay to operative management may increase the risk of adverse outcomes [9].

Triaging of emergency surgical cases were introduced at GSH for the categorization and prioritization of emergency surgical cases at the point of contact with the surgical team before the operative surgical management of a patient. The objective is to improve communication and teamwork between anesthetic, surgical, and nursing personnel involved in the care of these patients.

### Limitations

It is an observational study of a small number of trauma cases over 4-month period. The GSEST system describes the emergencies of all kinds including obstetric and gynecological. There are a few general surgical trauma diagnoses are included. It would be useful to have few more parameters perhaps in a separate column, e.g., what general surgical trauma diagnoses fit into the different category?

### Future directions

Every minute is essential for the severely injured trauma patient. Delays at any stage from the scene to the operating theater may impact negatively on the outcome regarding complications and death [9]. Triaging is a crucial element for such cases. A more extensive comparative research is required before and after the introduction of GSEST system to look at the effectiveness including over- and under-triage rate, accuracy, likelihood ratios, etc.

## Conclusion

Emergency trauma surgical cases vary in degree of acuity and complexity. Categorization allows the surgical and anesthetic teams to plan perioperative optimization and prioritize treatment of patients based on their clinical status and expected a progression of the disease. Assessing the South Africa’s excessive rate of penetrating wounds needing an emergency operation, it is of utmost importance to create, implement, use, and disseminate national guidelines and standard for these to improve outcomes and life losses.
